# New Tools for Health: COMUNI Questionnaire to Measure Dietary Quality of University Menus

**DOI:** 10.3390/nu17243873

**Published:** 2025-12-11

**Authors:** Beatriz de Mateo Silleras, Laura Carreño Enciso, Sandra de la Cruz Marcos, Emiliano Quinto Fernández, Paz Redondo del Río

**Affiliations:** Department of Nutrition and Food Science, Faculty of Medicine, University of Valladolid, 47005 Valladolid, Spain; bmateo@uva.es (B.d.M.S.); laura.carreno@uva.es (L.C.E.); equinto@uva.es (E.Q.F.); paz.redondo@uva.es (P.R.d.R.)

**Keywords:** questionnaire, university menu, food services, university students, dietary quality, diet, dietetics

## Abstract

**Background/Objectives**: The university stage is a critical period for consolidating dietary habits that influence future health. University canteens therefore play a key role in providing menus aligned with nutritional recommendations. As menu composition shapes students’ access to healthy food, its evaluation also has equity implications. This study aimed to apply a newly designed questionnaire—the COMUNI questionnaire—intended to provide a rapid, user-friendly, and transferable method for evaluating the dietary quality of lunch menus offered in university canteens. **Methods:** Two versions of the 13-item COMUNI questionnaire were developed: COMUNI-1 for single-option menus and COMUNI-2 for menus offering multiple first- and second-course choices. The tool evaluates the frequency of key food groups, the availability of water and wholegrain bread, and the variety of foods and culinary techniques. To test the questionnaire, it was applied to 34 menu templates from university residences, colleges, and cafeterias. **Results:** 85.3% of menus showed deficient dietary quality, and 14.7% were rated as improvable; none achieved an optimal score. Menus managed by catering companies obtained significantly higher scores than those under direct management. Most frequently shortcomings included insufficient offerings of vegetables, legumes, fish, and wholegrain bread, alongside a frequent presence of refined carbohydrate sources and fried or ultra-processed foods. **Conclusions:** Universities should incorporate adherence to dietary recommendations as a key criterion in food-service procurement. The COMUNI questionnaire provides a simple and operational tool for assessing menu quality, supporting both diagnosis and monitoring of university food-service, once formally validated. Its use may also help identify structural disparities in access to healthy foods across campus settings, supporting more equitable food-service policies.

## 1. Introduction

The early years of adulthood are crucial for establishing dietary habits that will persist in later stages of life, with consequent risks or benefits for health [1]. This life stage offers a key opportunity for health promotion and the reinforcement of healthy lifestyles [2]. However, several studies have documented that young adults often adopt diets of poor nutritional quality, characterized by low intake of plant-based foods and dairy products, and excessive consumption of sugary beverages, fast food, and snacks [2]. These habits are frequently accompanied by low physical activity levels and high alcohol intake [1]. Spanish university students exhibit these same trends [3], increasingly adopting a Western dietary pattern. Such unhealthy eating behaviours are associated with adverse health outcomes, even during young adulthood, including increased risk of overweight, obesity, mental health issues, and chronic diseases such as diabetes and cardiovascular disease [1,4]. Notably, weight gain during early adulthood has been identified as a strong predictor of later-life chronic disease risk [5].

The transition to university life often entails significant changes in eating habits. Many students leave the family home—where meals are usually planned and prepared by parents—and must begin to manage their own diet or rely on institutional food services such as residence halls or university cafeterias [1,6]. For many, these collective catering systems become their primary source of daily meals. In this context, although offering healthier menus does not automatically guarantee healthy eating habits, it does ensure the availability of healthier food options. Consequently, the quality and composition of the food provided on campus can substantially influence students’ dietary behaviours [7]. However, university canteens frequently offer a limited variety of foods and may lack affordable, health-promoting options [6], which can reinforce suboptimal eating patterns.

A growing body of evidence shows that food environments that ensure the availability, accessibility, and affordability of healthy foods facilitate healthier choices and support the establishment of adequate dietary habits [8,9,10,11]. In university settings, where many students rely on institutional food services for their main meals, limited availability of healthy options or higher prices for foods such as fruits and vegetables can act as structural barriers to healthy eating, contributing to inequities in access to nutritious foods [12,13]. Strengthening the nutritional quality and affordability of on-campus food provision is therefore essential to ensure that healthier options become the most accessible and convenient choices [14], thereby supporting broader public health objectives such as reducing diet-related non-communicable disease risk in line with Sustainable Development Goal 3.4.

Universities, therefore, play a strategic role in promoting healthy eating, both by ensuring the availability of healthy food options on campus and by implementing nutrition education initiatives [2]. Guaranteeing that university food services provide menus aligned with evidence-based dietary recommendations is essential. Nevertheless, traditional methods for assessing menu quality are time-consuming and require specialised personnel, which limits their routine application.

Over the past two decades, several tools have been developed to assess food-service environments and the nutritional quality of menus; however, most are not specifically tailored for university canteens. Early instruments, such as the Nutrition Environment Measures Survey in Restaurants (NEMS-R), focused primarily on environmental characteristics rather than menu composition itself [15]. Later, the Healthy Meal Index provided a simple nutrient-based assessment of meals in canteen settings but was developed outside the university context [16]. The FRESH audit expanded evaluation to environmental and behavioural supports within dining facilities [17]. More recent tools, such as AVACARD [18] and instruments assessing adherence to dietary patterns such as the Mediterranean Diet [19], offer structured menu evaluations, but remain oriented toward general food-service operations or specific dietary models. One of the few tools specifically designed for university or work-site canteens is the Nutrition Environment Scoring for Chinese-Style Canteens (NESC-CC), which incorporates culturally specific dietary characteristics [20]. Additionally, the Menu Assessment Scoring Tool (MAST) was developed to evaluate the nutritional quality of menus across diverse food-service establishments, although it is not tailored to university settings or Mediterranean dietary frameworks [21]. Collectively, these instruments often require detailed nutritional analyses, focus on broader food-environment factors, or target non-university populations, rather than providing a rapid and practical measure of menu quality.

Given these limitations, there remains scope for a brief and practical tool capable of evaluating the dietary quality of lunch menus in university canteens. Although developed within the Spanish higher-education context, such an instrument could also be adapted to other Mediterranean and European settings, where dietary recommendations and menu structures share common principles, and can be incorporated with minimal contextual modifications. Building on previous work developing the COMES questionnaire for the assessment of school menus [22], the present study applied a newly designed questionnaire intended to provide a rapid, user-friendly, and transferable method for evaluating the dietary quality of lunch menus offered in university canteens. However, the objective of this study was not to conduct a formal validation of the tool; as COMUNI is still in its pre-validation phase, the analyses presented should be interpreted solely as an initial demonstration of feasibility.

## 2. Materials and Methods

The COMUNI questionnaire (from the Spanish initials for university canteens—COMedores UNIversitarios) was developed to evaluate the dietary quality of lunch menus offered in university canteens. The validation of content was carried out through a review of national and international dietary recommendations [23,24,25,26] and a panel of experts. First, the selection of items was specifically based on the Food-Based Dietary Guidelines (FBDGs), as these provide science-based recommendations presented as practical guidelines for healthy eating [25,27,28,29]. Subsequently, the questionnaire underwent expert validation by the panel of experts in nutrition and dietetics, who assessed the clarity, relevance, and suitability of its items for the university context. During this process, experts also evaluated the adequacy of the items in relation to these recommendations, the appropriateness of the cut-off points used to assess each item, and the suitability of the final questionnaire classification based on the total score. As detailed in the Introduction, the present study does not include the psychometric validation of the tool; accordingly, the methodological focus here to describing its development and application.

COMUNI questionnaire consists of 13 items (Table 1). The first eight items assess the recommended frequency of consumption (FC) of the food groups typically eaten at the main meal in the Spanish context (lunch). These groups include vegetables, legumes, pasta, rice, potatoes, fish, meat, eggs, fruits, ultra-processed or pre-cooked, processed meat products (sausages and derivatives), and high-fat meats (such as chops, ribs, meatballs, or hamburgers). These items evaluate whether the menu offering aligns with the recommended consumption frequency for each food group. In addition, the questionnaire evaluates complementary aspects of the menu, such as whether wholegrain bread is available (item 9), whether water is offered as the default beverage (item 10), the variety of foods and culinary preparations, and the adequacy of cooking techniques used.

Two versions of the COMUNI questionnaire were proposed: (1) COMUNI-1, designed for menus offering a single fixed option (no choice available), and (2) COMUNI-2, intended for menus that provide two or more choices for both the first and second courses, from which users must select one of each (Table 1). Both versions are specifically designed to assess lunch menus served five days per week. The differences between COMUNI-1 and COMUNI-2 lie in five items (1, 2, 4, 5, and 6) for which the required frequencies vary. For example, COMUNI-2 requires higher weekly offerings of fish and legumes, given the availability of multiple dish options. In menus where salad, pasta, or potatoes are freely available—independently of the planned first or second course options—these foods are considered side dishes.

Each item is scored with 1 point if the criterion (the recommendation) is met, and 0 points if it is not. This information is obtained through a qualitative analysis of the menu template, considering the frequency of food group offerings, the variety of foods and preparations, and the cooking techniques used. To achieve this, the monthly or quarterly menu plan for each centre is reviewed and, when available, complemented with dish sheets specifying ingredients and quantities. Based on recommended portion sizes for this population [29], menu dishes are converted into servings allocated to the corresponding food groups, allowing the calculation of the weekly frequency of consumption for each group.

Items 1–8 are scored based on the weekly frequency with which each food group is offered. In the case of vegetables, first courses and side dishes are assessed separately, as lunch is the main meal in Spain and typically consists of a first course based on vegetables, legumes, cereals (e.g., pasta, rice), or potatoes; a protein-based second course (e.g., meat, fish, or eggs) accompanied by a side dish of vegetables, potatoes, or cereals to complement; and a dessert, often fruit, dairy, or sweets. Pre-cooked or ultra-processed foods include both fried pre-cooked items (e.g., croquettes, empanadas, calamari) and non-fried items (e.g., pizza, lasagne). Meats are classified into lean, fatty, and processed categories based on their distinct nutritional profiles and corresponding dietary guidelines. Other food groups were not included in this part of the questionnaire because they are indirectly assessed. For example, at lunchtime, dairy products and sweets are often served as dessert, displacing fruit consumption. Thus, compliance with item 3 of the COMUNI tool (“The menu offers fresh fruit every day”) implies the absence of dairy or sugar-based desserts. Likewise, adherence to item 10 (‘Water is the beverage offered with the menu’) excludes the provision of sugary or alcoholic drinks.

The COMUNI questionnaire also assesses the variety of both food items (i.e., offering foods from all groups and different items within the same group) and culinary techniques (evaluated both across the menu as a whole and within individual food groups) as additional indicators of menu quality. The evaluation criteria for food and culinary variety (Table 2) were established based on the dietary recommendations previously described and on nearly 20 years of experience from our research group in analysing menus from schools, universities, and other collective catering settings. For each food group, variety can be classified as “optimal,” “improvable,” or “deficient,” depending on the number of distinct foods or techniques offered per month. Cut-off points were defined according to the typical range of foods and preparation methods used in collective catering in Spain [30]. For example, (1) an optimal variety within the “pasta, rice, and potatoes” group requires the presence of all three foods; (2) likewise, three different legumes (traditionally lentils, chickpeas, and beans in Spain) are considered optimal for legumes group, and (3) six or more different vegetables are required given usual practice in institutional food services, even though many more exist. Similar criteria were applied to lean meats and fish. Cut-off points for culinary technique variety were based on the methods most commonly used in collective catering for each food group. When either the specific food item or the culinary technique used in a dish is not specified, that aspect is classified as “deficient.”

Item 11 evaluates the presence of fried, breaded, and battered foods on the menu; “fried” also includes mixed culinary preparations where frying is one of the cooking methods (e.g., Spanish omelet, meatballs). Item 12 is scored as zero if the variety of vegetables, legumes, meats, and fish is rated as “deficient” in more than one of these categories (Table 2). Item 13 is scored as zero if the variety of culinary techniques applied to vegetables, pasta, rice, potatoes, meats, and fish is rated as “deficient” in more than one category (Table 2).

The final score of the questionnaire is obtained by summing the scores of the 13 items. Menu dietary quality is classified as “deficient” when the total score ranges from 0 to 5 points (below the 33rd percentile), indicating that the recommendations are not met; “improvable” when the score is between 6 and 9 points (between the 33rd and 67th percentiles), indicating partial compliance with the recommendations; and “optimal” when the score is between 10 and 13 points (above the 67th percentile), indicating that most recommendations are fulfilled.

The implementation time of the COMUNI questionnaire is approximately 10 min. This tool has been developed for use both in the global assessment of university menus and in the identification of specific shortcomings through item-by-item evaluation.

### 2.1. Implementation of the COMUNI Questionnaire

To test the applicability and use of the designed tool, the COMUNI questionnaire was applied to menus from various Spanish university centres (residences, colleges, and cafeterias). Menu plans were obtained either by direct request or online, and all menu templates to which access was possible—provided they met the inclusion criteria—were analysed. Inclusion criteria required a minimum of 3–4 weeks of planned menus and affiliation with a university dining service. The purposeful sample of templates employed was selected to reflect the diversity of menu structures, dish-choice systems, and management models commonly found in university canteens. Menus were prepared and served under two management models: (1) “direct management”, in which the menu planning and food preparation were carried out internally by the institution or by small-scale catering providers, and (2) “catering”, in which the service was outsourced to food service management companies. The qualitative analysis of the menu sheets was conducted as previously described, and the COMUNI-1 or COMUNI-2 questionnaires were administered accordingly, based on the menu options.

### 2.2. Statistical Analysis

The results are presented as mean (SD) or absolute frequency (n). The assessment of individual questionnaire items is expressed as the percentage of positive responses (% compliance with recommendations). The normality of quantitative variables was evaluated using the Shapiro–Wilk test. As the questionnaire score did not follow a normal distribution, comparisons were performed using the Mann–Whitney U test (for dichotomous variables) or the Kruskal–Wallis H test (for variables with more than two categories). Categorical variables were compared using the Chi-square test. Statistical significance was set at *p* < 0.05. Statistical analyses were conducted using IBM SPSS version 29.0.

## 3. Results

The COMUNI questionnaire was applied to 34 menu sheets from university canteens across seven Spanish regions. As the tool is still in a pre-validation phase and the sample was small and non-random, the results presented should be interpreted as descriptive and exploratory. These menus were served in 9 university residences, 12 colleges, and 13 university cafeterias. In terms of food service management, 15 menus were provided by food service management companies (4 from residences, 9 from student halls, and 2 from cafeterias), while 19 were directly managed by the institution or by small-scale catering providers (5 residences, 3 student halls, and 11 cafeterias). Of the 34 menus, 12 offered no dish choice and were therefore assessed using COMUNI-1. In 13 menus, two options were available for both the first and second courses; in the remaining 9, three or more options were offered. These 22 menus with multiple options were evaluated using COMUNI-2.

Table 3 shows the mean scores of the COMUNI, COMUNI-1, and COMUNI-2 questionnaires for the overall sample, as well as by type of university centre and food service management model. No statistically significant differences were observed between COMUNI-1 and COMUNI-2 scores (*p* = 0.307), nor across different types of university centres (*p* = 0.329). However, menus managed by food service management companies scored significantly higher than those overseen by institutions or small-scale catering providers (*p* < 0.001). This difference remained significant when analysing COMUNI-1 (*p* = 0.004) and COMUNI-2 (*p* = 0.021) separately. Regarding the classification of the questionnaires, none of the analysed menus achieved an “optimal” rating (10–13 points).

Table 4 shows the distribution of menus classified as having either “poor” (P) (0–5 points) or “improvable” (I) (6–9 points) dietary quality. No significant differences were found in classification between COMUNI, COMUNI-1, and COMUNI-2 or across types of university centres and food service management.

Finally, individual questionnaire items were analysed (Table 5). Results showed that most canteens served less fish, legumes, vegetables (both as main and side dishes), and whole-grain bread than recommended. Conversely, there was an excessive presence of pasta, rice, or potatoes as first courses, processed and fatty meats, and fried, breaded, or battered preparations. However, water was consistently offered as the default beverage, and fresh fruit was present as dessert in most menus. The variety of culinary techniques was generally improvable. Menus managed by catering companies performed significantly better in two key areas: they included fewer precooked and ultra-processed foods (*p* = 0.026) and offered a greater variety of foods within the same group (*p* = 0.006), compared to menus under direct management. These findings illustrate the type of patterns the tool can detect in practice.

## 4. Discussion

The present study developed and applied two brief versions of the COMUNI questionnaire (COMUNI-1 and COMUNI-2) to assess the dietary quality of lunch menus offered in university canteens. The tool was conceptualized based on food-based dietary recommendations for the Spanish population [25,27], the Mediterranean dietary pattern [28], and existing evidence documenting suboptimal eating habits among university students [1,31]. Rather than evaluating nutrient composition, COMUNI focuses on the frequency and presence of key food groups and culinary practices, consistent with the approach followed by other menu-assessment indices [32]. This methodological process aimed to produce a user-friendly tool that could be completed rapidly, enabling the routine and operational assessment of university menus. Accordingly, the results presented here should be understood as descriptive and illustrative of the tool’s potential operational use.

Although several instruments have been developed to assess food-service environments and menu quality, most were not designed for university settings and therefore have limited applicability to this population. Early tools, such as the Nutrition Environment Measures Survey in Restaurants (NEMS-R), which focuses primarily on restaurant environments, do not directly evaluate the structure of institutional menus [15]. Similarly, the Healthy Meal Index was validated in non-university canteens [16], and the Full Restaurant Evaluation Supporting a Healthy dining environment (FRESH) expands assessment to environmental and behavioural supports without specifically analysing menu composition [17]. More recent menu-based instruments—such as AVACARD [18] or tools designed to assess adherence to the Mediterranean diet in cafeteria menus [19]—offer structured evaluations but remain oriented toward general food-service operations or to specific dietary patterns.

Within this heterogeneous landscape, the Menu Assessment Scoring Tool (MAST), developed in Australia, represents an additional attempt to evaluate the nutritional quality of menus across diverse food-service establishments [21]. However, MAST was conceived for broad application and not specifically for higher-education institutions. As its authors acknowledge, it provides an overall appraisal of the availability of nutritious and nutrient-poor foods and beverages but does not assess the adequacy of individual dishes or adherence to specific dietary patterns, thus requiring complementary and more detailed analyses when an in-depth menu evaluation is needed. Even the Nutrition Environment Scoring for Chinese-Style Canteens (NESC-CC), one of the few tools specifically developed for university or work-site canteens, is tailored to Chinese-style menus and incorporates culturally specific scoring criteria [20].

School-menu assessment tools have also been developed to evaluate menu quality [33,34,35,36], but they also present limited transferability, as they target the nutritional needs and eating behaviours of children rather than young adults. Systematic reviews highlight this heterogeneity and the limited cross-context applicability of existing menu-assessment indices [32], reinforcing the need for adaptable, population-specific tools.

In this context, COMUNI provides a brief, food-based instrument tailored to the characteristics of university lunch menus and adaptable to Mediterranean and broader European settings. Although developed within the Spanish context, its structure—organized around food groups, menu frequency criteria, and basic culinary practices—facilitates its application in other countries whose food-based dietary guidelines share similar principles. Across the European Union, national FBDGs consistently emphasise core elements such as regular consumption of vegetables, fruits, whole grains, legumes, fish, and water, while discouraging foods high in saturated fats, sugars, and salt [23,37]. Given this common foundation, COMUNI can be readily adapted to diverse European food-service contexts with minimal contextual modifications. Moreover, the tool can be applied in approximately 10 min, allowing for rapid assessment without compromising feasibility, whereas other authors, such as Sealens (NEMS-R tool) [15], have reported application times of around 30 min for their questionnaire.

In this study, COMUNI was applied to 34 menus from Spanish university residences, colleges, and cafeterias. The objective was not to evaluate the overall quality of university menus at the national level, but rather to test the performance and applicability of the tool. Although the results obtained from our sample cannot be considered representative of all university food services, they reveal a pattern consistent with previous research: the availability of food groups that students typically underconsume—such as vegetables, legumes, fruits, and fish—is limited, whereas items that young adults tend to overconsume, including pasta, rice, potatoes, processed meats, and fried foods, are more frequently offered [2,38,39]. Consequently, menu compliance with the recommended frequencies for these key food groups was generally low. The high prevalence of energy-dense, low-nutrient options and the limited presence of plant-based foods are aligned with dietary patterns previously reported among Spanish university students, who show low adherence to healthy dietary models and suboptimal overall diet quality [40,41].

From the perspective of the Mediterranean diet—considered a protective dietary model characterized by high consumption of vegetables, legumes, fruits, nuts, and olive oil; moderate intake of dairy, eggs, and fish; and limited consumption of meat and processed foods [42]—our results indicate low adherence among the assessed menus. Specifically, the reduced frequency of legumes, fish, and vegetables and the predominance of refined carbohydrate sources and fried preparations are inconsistent with Mediterranean dietary principles. These findings are consistent with recent concerns regarding declining adherence to the Mediterranean diet among young adults in Mediterranean countries [43].

The menus prepared and served by catering companies scored significantly higher than those under direct management. Although differences did not always reach statistical significance—likely due to sample size—catered menus were more aligned with dietary recommendations across several items, including a lower presence of ultra-processed products, a greater diversity of protein sources, and more frequent provision of fruit as dessert. This may reflect the more standardised procedures, and the greater involvement of nutrition professionals typically present in catering services in our country—Spanish regulations require registered dietitians only in catering companies, but not in smaller food-service providers—a trend also observed in previous studies on institutional food service quality [6,7].

University canteens have a key role in shaping the food environment and facilitating healthy eating behaviours [6,7]. Several strategies have been devised to promote healthy eating in this population group, such as providing nutritional information on menus (energy, nutrients, fat) or implementing some form of front-of-pack food labelling system (nutritional traffic light, health seals, etc.) [6,44], with the latter generally proving more effective in guiding students toward healthier selections. However, many university canteens offer a single menu option, limiting the applicability of choice-based interventions. In such contexts, ensuring that all menus adhere to nutritional recommendations becomes essential. Conversely, in settings where multiple options are available, labelling and informational strategies can serve as useful tools to support students in choosing healthier dishes. The consistently low COMUNI scores observed across our menu template sample from different institutions and regions indicate a substantial margin for improvement in the nutritional quality of menus. Enhancing the presence of vegetables, legumes, whole grains, and fish, while reducing processed meats and fried foods, would better align university offerings with national and international dietary recommendations [25,26,28]. Complementary strategies—such as providing nutritional information, incorporating front-of-pack labelling systems, and implementing nutrition education initiatives—can further reinforce healthy eating behaviours and support universities in their commitment to fostering long-term student wellbeing.

Beyond their nutritional implications, these findings are relevant from an equity perspective. When daily menus systematically lack healthy, diverse, and affordable options, students’ ability to choose nutritionally adequate meals becomes structurally constrained, regardless of individual motivation or knowledge. In this sense, the low COMUNI scores observed across the sample point not only to suboptimal menu planning but also to potential disparities in access to healthy food within university settings. Strengthening the healthfulness and consistency of menu offerings is therefore essential to ensure that all students—particularly those who rely on campus dining as their primary food source—can access and choose healthier options on an equitable basis.

The present study has several limitations. The first and most important is that the questionnaire needs to be validated before it can be used in research studies. The aim of our study was not to validate the tool, but rather to apply a newly designed questionnaire to illustrate its feasibility, usability, and the type of information it can generate when assessing the dietary quality of lunch menus in university canteens. Future work will focus on completing the validation process, including reliability testing and evaluation of criterion validity. The proposal of the COMUNI questionnaire should therefore be understood as an initial framework that may also inspire the development of similar tools in other countries or contexts.

Additionally, the menu sample analysed was small and non-random, which prevents generalisation of the findings to all Spanish university food services. However, the purpose of the menu analysis was illustrative rather than inferential, aiming to demonstrate how the tool can be applied, how results are interpreted, and how the instrument can help identify areas of non-compliance with dietary recommendations.

COMUNI may serve as an operational diagnostic tool to support universities in monitoring and improving the nutritional quality of their food services, once it undergoes a formal validation process. Its structured, item-based assessment enables the identification of specific weaknesses and allows periodic monitoring, making it useful not only for internal quality improvement but also for verifying compliance with the dietary standards established in procurement specifications. In this regard, universities should explicitly incorporate adherence to dietary recommendations as a key criterion in the awarding and renewal of food-service contracts across all centres [45], ensuring that catering providers meet the required nutritional criteria.

In our study, none of the evaluated menus achieved an “optimal” (10–13 points) classification. Although scoring thresholds may be demanding, aligning menus with dietary guidelines is essential if universities are to fulfil their role as health-promoting institutions [1] and contribute to reducing the burden of diet-related chronic diseases [25,27,28]. Tools such as COMUNI can therefore facilitate oversight, accountability, and continuous improvement in university food-service provision.

## 5. Conclusions

The COMUNI questionnaire is a quick and easy tool for assessing the dietary quality of university lunch menus. By evaluating the frequency of essential food groups, the use of culinary techniques, and the variety of foods offered enables both an overall assessment of menu quality and the identification of specific areas needing improvement through item-level analysis. Given the role of universities as health-promoting institutions, adherence to dietary recommendations should be incorporated as a key criterion when awarding food service contracts across all university centres [45]. Once further validated, the COMUNI questionnaire can support this process by providing a structured and operational framework for menu quality assessment, while its periodic application allows for the longitudinal monitoring of university food services. In addition, by revealing systematic gaps in the availability of healthy food groups, COMUNI may help identify structural barriers within campus dining services, thereby supporting efforts to improve equitable access to healthier food options for all students.

Ultimately, COMUNI could not only contribute to improving students’ nutritional environments but also advance broader goals related to public health and food system sustainability. Complementary strategies—such as providing nutritional information, introducing front-of-pack labelling systems, and implementing nutrition education—are also needed to reinforce healthy eating habits and support universities in their commitment to promoting long-term wellbeing.

## Figures and Tables

**Table 1:** COMUNI-1 and COMUNI-2 questionnaires for dietary assessment of the quality of university menus without options for dish selection.

Items COMUNI-1	Points	Items COMUNI-2	Points
1. The menu offers fish at least three times a week		1. The menu offers fish at least four times a week.	
2. The menu offers legumes at least twice a week.		2. The menu offers legumes at least three times a week. ^1^	
3. The menu offers fresh fruit every day.		3. The menu offers fresh fruit every day.	
4. The menu offers vegetables as a first course at least twice a week.		4. The menu offers vegetables as a first course every day.	
5. The menu offers a side dish of vegetables at least four times a week.		5. The menu offers a side dish of vegetables every day.	
6. The menu offers potatoes, pasta, or rice as a first course at most once a week.		6. The menu offers potatoes, pasta, or rice as a first course up to twice a week at most.	
7. The menu offers pre-cooked or ultra-processed foods at most once every two weeks.		7. The menu offers pre-cooked or ultra-processed foods at most once every two weeks.	
8. The menu offers processed meats or fatty meats at most once every two weeks.		8. The menu offers processed meats or fatty meats at most once every two weeks.	
9. Whole-grain bread is offered.		9. Whole-grain bread is offered	
10. The beverage offered on the menu is water.		10. The beverage offered on the menu is water.	
11. The presence of fried, breaded, and battered foods on the menu is less than or equal to once a week.		11. The presence of fried, breaded, and battered foods on the menu is less than or equal to twice a week.	
12. There is an adequate variety of foods within the same group.		12. There is an adequate variety of foods within the same group.	
13. There is an adequate variety of culinary preparations, both across the menu in general and for the same foods.		13. There is an adequate variety of culinary preparations, both across the menu in general and for the same foods.	

Each item is scored 1 point if it meets the criterion, or 0 points if it does not. ^1^ For menus with more than 2 options for each course, this food group must be offered every day.

**Table 2:** Assessment criteria to monthly evaluate the variety of foods and culinary techniques of university menus with the COMUNI questionnaire.

Course	Food Group	Evaluation	Variety Within the Group	Variety of Culinary Techniques
First course	Pasta, rice, and potatoes	Optimal	3	4
		Improvable	2	3
		Deficient	1	2
	Vegetables	Optimal	≥6	≤4
		Improvable	4–5	2–3
		Deficient	≤3	1
	Legumes	Optimal	3	3
		Improvable	2	2
		Deficient	1	1
Second course	Lean meats	Optimal	4	≥4
		Improvable	3	3
		Deficient	≤2	≤2
	Fish	Optimal	≥5 (b and a)	≥4
		Improvable	3–4 (b and a)	2–3
		Deficient	≤2 (or no b and a)	1
Side dish	Vegetables	Optimal	≥3	3
		Improvable	2	2
		Deficient	1	1
	Potatoes	Optimal		3
		Improvable		2
		Deficient		1

a: oily fish; b: white fish.

**Table 3:** Mean scores of the COMUNI questionnaire and the COMUNI-1 and COMUNI-2 questionnaires for the total sample and by type of university centre and food service management.

Centers		COMUNI Mean (SD)	COMUNI-1 Mean (SD)	COMUNI-2 Mean (SD)
Total sample		n = 34 4.3 (1.4)	n = 12 3.9 (1.2)	n = 22 4.5 (1.5)
Type of university centre	University residence	n = 9 3.8 (1.1)	n = 6 3.5 (1.0)	n = 3 4.3 (1.2)
	Student hall	n = 12 4.7 (1.2)	n = 6 4.3 (1.2)	n = 6 5.0 (1.3)
	University cafeteria	n = 13 4.3 (1.7)	n = 0	n = 13 4.3 (1.7)
Management modality	Catering	n = 15 5.1 (1.2) *	n = 6 4.8 (0.8) *	n = 9 5.3 (1.4) *
	Direct management	n = 19 3.6 (1.2)	n = 6 3.0 (0.6)	n = 13 2.9 (1.3)

* *p* < 0.05 Catering vs. Direct management. Classification ranges: poor = 0–5 points; improvable = 6–9 points; optimal = 10–13 points.

**Table 4:** Classification of the COMUNI questionnaire and the COMUNI-1 and COMUNI-2 questionnaires for the total sample and by type of university centre and food service management.

COMUNI Classification		COMUNI		COMUNI-1		COMUNI-2	
		P	I	P	I	P	I
Total sample		29	5	11	1	18	4
Type of university centre	University residence	9	0	6	0	3	0
	Student hall	9	3	5	1	4	2
	University cafeteria	11	2	0	0	11	2
Management modality	Catering	11	4	6	1	6	3
	Direct management	18	1	6	0	12	1

P: poor dietary quality (0–5 points); I: improvable dietary quality (6–9 points). No menus achieved an optimal score (10–13 points).

**Table 5:** Compliance with COMUNI questionnaire items for the total sample and according to management modality.

Compliance with COMUNI Items (%)	COMUNI	Management Modality	
		C	DM
1. Fish	29.4	33.3	26.3
2. Legumes	11.8	20.0	5.3
3. Fresh fruit	94.1	93.3	94.7
4. Vegetables as a main dish	32.4	33.3	31.6
5. Vegetables as side dish	41.2	46.7	36.8
6. Pasta, potatoes and rice as a main dish	2.9	6.7	0
7. Pre-cooked or ultra-processed foods	58.8	80.0 *	42.1
8. Processed meats or fatty meats	0	0	0
9. Whole-grain bread	5.9	13.3	0
10. Water	100	100	100
11. Fried, breaded, and battered foods	5.9	13.3	0
12. Variety of foods within the same group	47.1	73.3 *	26.3
13. Variety of culinary preparations	70.6	80.0	63.2

* *p* < 0.05 Catering vs. Direct management. C: catering; DM: direct management.

## Data Availability

The original contributions presented in this study are included in the article/Supplementary Material. Further inquiries can be directed to the corresponding author.

## Supplementary Materials

The following supporting information can be downloaded at: https://www.mdpi.com/article/10.3390/nu17243873/s1, Table S1: Qualitative analysis of the studied menu sheets; Table S2: COMUNI questionnaire results of the studied menu sheets.

## Abbreviations

The following abbreviations are used in this manuscript:

AVACARD	Menu Evaluation Index
C	Catering
COMUNI	Name of the proposed questionnaire; from the Spanish initials for Comedores Universitarios (university canteens)
DM	Direct management
FBDGs	Food-Based Dietary Guidelines
FC	Frequency of consumption
FRESH	Full Restaurant Evaluation Supporting a Healthy Dining Environment
I	Improvable
MAST	Menu Assessment Scoring Tool
NEMS-R	Nutrition Environment Measures Survey in Restaurants
NESC-CC	Nutrition Environment Scoring for Chinese-Style Canteens
P	Poor
SD	Standard deviation
SPSS	Statistical Package for the Social Sciences
WHO	World Health Organization

## References

[1] Almoraie N.M., Alothmani N.M., Alomari W.D., Al-Amoudi A.H. (2025). Addressing Nutritional Issues and Eating Behaviours among University Students: A Narrative Review. Nutr. Res. Rev..

[2] Mueller M.P., Blondin S.A., Korn A.R., Bakun P.J., Tucker K.L., Economos C.D. (2018). Behavioral Correlates of Empirically-Derived Dietary Patterns among University Students. Nutrients.

[3] Ministerio de Sanidad, Servicios Sociales e Igualdad, Instituto Nacional de Estadística (2017). Encuesta Nacional de Salud.

[4] World Health Organization (2014). Global Status Report on Noncommunicable Diseases 2014.

[5] Zheng Y., Manson J.E., Yuan C., Liang M.H., Grodstein F., Stampfer M.J., Willett W.C., Hu F.B. (2017). Associations of Weight Gain from Early to Middle Adulthood with Major Health Outcomes Later in Life. JAMA.

[6] Fogolari N., Souza A.D., Bernardo G.L., Uggioni P.L., Oliveira R.C., Rodrigues V.M., Proença R.P.C., Fernandes A.C. (2023). Qualitative Menu Labelling in University Restaurants and Its Influence on Food Choices: A Systematic Review and Synthesis without Meta-Analysis. Nutr. Bull..

[7] Fonseca L.B., Pereira L.P., Rodrigues P.R.M., Andrade A.C.S., Muraro A.P., Gorgulho B.M., Pereira R.A., Ferreira M.G. (2021). Food Consumption on Campus Is Associated with Meal Eating Patterns among College Students. Br. J. Nutr..

[8] Minaker L.M., Shuh A., Olstad D.L., Engler-Stringer R., Black J.L., Mah C.L. (2016). Retail Food Environments Research in Canada: A Scoping Review. Can. J. Public Health.

[9] Fergus L., Seals K., Holston D. (2021). Nutrition Interventions in Low-Income Rural and Urban Retail Environments: A Systematic Review. J. Acad. Nutr. Diet..

[10] Brimblecombe J., Miles B., Chappell E., De Silva K., Ferguson M., Mah C., Miles E., Gunther A., Wycherley T., Peeters A. (2023). Implementation of a Food Retail Intervention to Reduce Purchase of Unhealthy Food and Beverages in Remote Australia: Mixed-Method Evaluation Using the Consolidated Framework for Implementation Research. Int. J. Behav. Nutr. Phys. Act..

[11] Franchini C., Biasini B., Sogari G., Wongprawmas R., Andreani G., Dolgopolova I., Gómez M.I., Roosen J., Menozzi D., Mora C. (2024). Adherence to the Mediterranean Diet and Its Association with Sustainable Dietary Behaviors, Sociodemographic Factors, and Lifestyle: A Cross-Sectional Study in US University Students. Nutr. J..

[12] Li X., Braakhuis A., Li Z., Roy R. (2022). How Does the University Food Environment Impact Student Dietary Behaviors? A Systematic Review. Front. Nutr..

[13] Caruso O.T., Schaafsma H.N., McEachern L.W., Gilliland J.A. (2025). The Campus Food Environment and Post-Secondary Student Diet: A Systematic Review. J. Am. Coll. Health.

[14] Glanz K., Sallis J.F., Saelens B.E., Frank L.D. (2005). Healthy Nutrition Environments: Concepts and Measures. Am. J. Health Promot..

[15] Saelens B.E., Glanz K., Sallis J.F., Frank L.D. (2007). Nutrition Environment Measures Study in Restaurants (NEMS-R): Development and Evaluation. Am. J. Prev. Med..

[16] Lassen A.D., Biltoft-Jensen A., Hansen G.L., Hels O., Tetens I. (2010). Development and Validation of a New Simple Healthy Meal Index for Canteen Meals. Public Health Nutr..

[17] Horacek T.M., Yildirim E.D., Simon M., Byrd-Bredbenner C., White A.A., Shelnutt K.P., Olfert M.D., Morrell J., Mathews A., Kidd T. (2018). Development and Validation of the Full Restaurant Evaluation Supporting a Healthy (FRESH) Dining Environment Audit. J. Hunger Environ. Nutr..

[18] Lima L.B., Akutsu R.C.C.A., Botelho R.A., Nakano E.Y. (2023). AVACARD—Menu Evaluation Index: Construction and Validation. Int. J. Gastron. Food Sci..

[19] Silva S.S., Rocha A., Ferreira L., Neto B., Dikmen D., Filipec S.V., Satalic Z., Viegas C. (2024). Development of a Tool to Assess the Compliance of Cafeteria Menus with the Mediterranean Diet. BMC Nutr..

[20] Han Y., Fan Z., Wu Y., Zhang D., Wen X. (2022). Development and Validation of Nutrition Environment Scoring for Chinese-Style University/Work-Site Canteens (NESC-CC) and Oil-Salt Visual Analogue Scale (OS-VAS). Int. J. Environ. Res. Public Health.

[21] Pulker C.E., Aberle L.M., Butcher L.M., Whitton C., Law K.K., Large A.L., Pollard C.M., Trapp G.S.A. (2023). Development of the Menu Assessment Scoring Tool (MAST) to Assess the Nutritional Quality of Food Service Menus. Int. J. Environ. Res. Public Health.

[22] De Mateo Silleras B., Martín M.A.C., Sainz B.O., Enciso L.C., De la Cruz Marcos S., De Miguelsanz J.M.M., Del Río P.R. (2015). Design and Implementation of a Questionnaire to Evaluate the Dietary Quality of School Meals. Nutr. Hosp..

[23] FAO Dietary Guidelines. https://www.fao.org/nutrition/education/food-dietary-guidelines/home/en/.

[24] EFSA Your Nutrition Needs. https://www.efsa.europa.eu/en/safe2eat/your-nutrition-needs.

[25] Agencia Española de Seguridad Alimentaria y Nutrición (AESAN) (2022). Recomendaciones Dietéticas Saludables y Sostenibles con Recomendaciones de Actividad Física Para la Población Española: Come Sano, Muévete y Cuida tu Planeta.

[26] Cámara M., Giner R.M., González-Fandos E., López-García E., Mañes J., Portillo M.P., Rafecas M., Domínguez L., Martínez J.A. (2021). Food-Based Dietary Guidelines around the World: A Comparative Analysis to Update AESAN Scientific Committee Dietary Recommendations. Nutrients.

[27] Aranceta-Bartrina J., Partearroyo T., López-Sobaler A.M., Ortega R.M., Varela-Moreiras G., Serra-Majem L., Pérez-Rodrigo C. (2019). Updating the Food-Based Dietary Guidelines for the Spanish Population: The Spanish Society of Community Nutrition (SENC) Proposal. Nutrients.

[28] Serra-Majem L., Tomaino L., Dernini S., Berry E.M., Lairon D., De la Cruz J.N., Bach-Faig A., Donini L.M., Medina F.X., Belahsen R. (2020). Updating the Mediterranean Diet Pyramid towards Sustainability: Focus on Environmental Concerns. Int. J. Environ. Res. Public Health.

[29] SENC–GLANC (2025). Guías Alimentarias de la SENC para la Población Española. Nuevas Pirámides SENC 2025/26. Rev. Esp. Nutr. Comunitaria.

[30] Ministerio de Agricultura, Pesca y Alimentación de España (MAPA) Panel de Consumo Alimentario. https://www.mapa.gob.es/es/alimentacion/temas/consumo-tendencias/panel-de-consumo-alimentario.

[31] Sprake E.F., Russell J.M., Cecil J.E., Cooper R.J., Grabowski P., Pourshahidi L.K., Barker M.E. (2018). Dietary Patterns of University Students in the UK: A Cross-Sectional Study. Nutr. J..

[32] Gorgulho B.M., Pot G.K., Sarti F.M., Marchioni D.M. (2016). Indices for the Assessment of Nutritional Quality of Meals: A Systematic Review. Br. J. Nutr..

[33] Cupertino A.F., Maynard D.C., de Queiroz F.L.N., Zandonadi R.P., Ginani V.C., Raposo A., Saraiva A., Botelho R.B.A. (2021). How Are School Menus Evaluated in Different Countries? A Systematic Review. Foods.

[34] Rocha A., Viegas C. (2020). KIMEHS—Proposal of an Index for Qualitative Evaluation of Children’s Menus: A Pilot Study. Foods.

[35] Krukowski R.A., Eddings K., West D.S. (2011). The Children’s Menu Assessment: Development, Evaluation, and Relevance of a Tool for Evaluating Children’s Menus. J. Am. Diet. Assoc..

[36] Llorens-Ivorra C., Quiles-Izquierdo J., Richart-Martínez M., Arroyo-Bañuls I. (2016). Diseño de un Cuestionario para Evaluar el Equilibrio Alimentario de Menús Escolares. Rev. Esp. Nutr. Hum. Diet..

[37] EFSA (2010). Scientific Opinion on Establishing Food-Based Dietary Guidelines. EFSA J..

[38] Salameh P., Jomaa L., Issa C., Farhat G., Salamé J., Zeidan N., Baldi I., Barbour B., Waked M., Zeghondi H. (2014). Assessment of Dietary Intake Patterns and Their Correlates among University Students in Lebanon. Front. Public Health.

[39] Bernal-Orozco M.F., Salmeron-Curiel P.B., Prado-Arriaga R.J., Orozco-Gutiérrez J.F., Badillo-Camacho N., Márquez-Sandoval F., Altamirano-Martínez M.B., González-Gómez M., Gutiérrez-González P., Vizmanos B. (2020). Second Version of a Mini-Survey to Evaluate Food Intake Quality (Mini-ECCA v.2): Reproducibility and Ability to Identify Dietary Patterns in University Students. Nutrients.

[40] Ramón-Arbués E., Granada-López J.M., Martínez-Abadía B., Echániz-Serrano E., Antón-Solanas I., Jerue B.A. (2021). Factors Related to Diet Quality: A Cross-Sectional Study of 1055 University Students. Nutrients.

[41] Martínez Álvarez J.R., García Alcón R., Villarino Marín A., Marrodán Serrano M.D., Serrano Morago L. (2015). Eating Habits and Preferences among the Student Population of the Complutense University of Madrid. Public Health Nutr..

[42] Serra-Majem L., Román-Viñas B., Sanchez-Villegas A., Guasch-Ferré M., Corella D., La Vecchia C. (2019). Benefits of the Mediterranean Diet: Epidemiological and Molecular Aspects. Mol. Asp. Med..

[43] Ejeda-Manzanera J.M., Rodrigo-Vega M. (2021). Hábitos de Alimentación y Calidad de Dieta en Estudiantes Universitarias de Magisterio en Relación con su Adherencia a la Dieta Mediterránea. Eating habits and diet quality in university students of teaching in relation to their adherence to the Mediterranean diet. Rev. Esp. Salud Pública.

[44] Cerezo-Prieto M., Frutos-Esteban F.J. (2021). Towards Healthy Pathways: Effect of Nutrition Labels on Eating Behaviours in a University Canteen. Aten. Primaria.

[45] Fandetti S.M., Dahl A.A., Webster C., Bably M.B., Coffman M.J., Racine E.F. (2023). Healthy Food Policies Documented in University Food Service Contracts. Int. J. Environ. Res. Public Health.

