# Supplementary material for: New Tools for Health: COMUNI Questionnaire to Measure Dietary Quality of University Menus
Source: Nutrients. 2025 Dec 11;17(24):3873. doi: 10.3390/nu17243873 (PMC12736357; doi:10.3390/nu17243873)
Supplement: Supplementary file 1 [file nutrients-17-03873-s001.zip › nutrients-4008428-supplementary.pdf]

SUPLEMENTARY MATERIAL

Table S1. Qualitative analysis of the studied menu sheets.

ID	Centre	Region	Management	Menu options	Frequency of consumption per week									Variety of foods within the group per month				Variety of culinary techniques per month			
					Fish	Legumes	Fruit	Vegetables	Side dish vegetables	Potatoes/ pasta/ rice	Pre-cooked/ ultra-processed	Processed/ fatty meat	Fried food	Vegetables	Legumes	Lean meats	Fish	Vegetables	Potatoes/pasta/ rice	Lean meats	Fish
1	2	7	2	3	4,8	2,5	5,0	5,9	1,0	10,1	3,0	3,4	3,8	11	3	3	5	6	12	2	8
2	2	3	2	2	5,1	1,5	5,0	5,0	1,3	3,5	0,5	1,3	2,5	12	3	4	9	7	9	3	11
3	1	1	1	1	1,5	1,5	5,0	1,3	2,3	2,4	0,5	1,1	1,5	2	3	2	6	2	12	5	6
4	2	3	1	1	1,1	0,9	5,0	1,0	0,3	3,3	0,0	1,3	2,3	3	3	3	2	3	11	4	1
5	3	4	1	2	5,1	1,1	5,0	4,9	0,5	3,3	1,0	1,9	3,5	12	2	3	0	9	7	3	0
6	3	1	1	3	5,1	3,5	5,0	15,6	10,0	6,4	0,5	2,4	4,3	21	3	5	8	9	18	10	13
7	1	7	2	3	3,9	1,3	5,0	8,0	0,8	5,6	1,5	2,3	2,0	5	2	3	6	5	13	4	7
8	2	3	2	1	1,0	2,0	5,0	1,3	3,5	2,0	0,0	2,4	0,8	4	3	3	4	5	8	3	4
9	1	3	1	1	1,1	1,3	5,0	0,8	5,0	3,5	0,0	1,5	0,8	3	3	5	3	3	12	13	3
10	1	2	2	1	2,0	1,5	5,0	0,0	5,0	3,5	0,5	1,0	1,8	0	3	1	0	0	9	2	0
11	1	1	1	1	1,0	1,3	3,8	1,0	2,8	2,8	0,5	1,4	2,8	3	2	3	1	3	8	3	2
12	1	1	1	1	1,0	2,0	5,0	0,5	2,5	2,8	0,8	2,0	2,0	2	3	1	4	2	9	2	3
13	1	3	2	2	2,0	3,3	5,0	3,0	3,8	4,0	0,5	3,3	1,5	7	3	5	2	9	14	7	8
14	1	5	2	1	1,0	2,3	5,0	1,0	6,3	3,0	0,0	1,4	3,0	2	3	4	3	2	9	7	3
15	2	3	1	1	1,8	1,3	5,0	0,3	3,3	3,5	0,3	1,9	2,0	1	3	3	3	1	10	3	6
16	3	5	2	2	4,8	2,0	5,0	5,5	5,8	3,0	0,4	2,9	3,5	8	3	5	11	15	11	11	15
17	3	6	2	2	4,4	2,0	5,0	3,8	3,0	4,1	0,0	3,3	1,8	8	3	3	7	8	15	4	13
18	3	7	1	3	4,8	3,3	5,0	8,3	0,0	12,5	3,5	4,3	9,5	8	3	5	11	4	28	10	18
19	3	3	1	2	2,3	1,0	5,0	3,0	5,0	6,1	1,5	3,6	3,8	8	2	3	3	8	14	4	4
20	3	3	1	2	1,5	0,5	5,0	4,3	5,0	4,9	2,3	3,0	4,5	9	1	3	2	8	15	5	3
21	3	3	1	2	1,8	0,5	5,0	4,5	5,0	5,4	2,8	3,1	4,8	16	2	3	4	14	10	4	5
22	3	3	1	2	2,1	1,8	5,0	3,3	5,0	4,9	4,0	2,1	5,5	7	3	1	4	4	9	4	4
23	3	3	1	3	4,3	2,5	5,0	7,0	0,0	5,1	4,3	5,4	4,8	5	3	5	5	6	11	3	10
24	2	5	2	2	1,0	1,5	1,5	7,0	1,8	3,3	0,0	0,7	2,5	6	3	6	4	14	9	9	4
25	3	3	1	3	5,1	3,5	5,0	5,3	0,0	7,3	5,0	9,1	6,5	4	3	1	7	7	12	3	6
26	2	3	2	1	0,5	1,8	5,0	0,8	5,0	2,6	0,0	2,4	2,0	3	3	5	2	3	10	5	2
27	2	3	2	1	1,1	1,5	5,0	1,0	5,0	2,5	0,0	2,3	2,8	4	3	5	4	3	10	5	3
28	2	3	2	1	1,5	1,3	5,0	1,3	5,0	2,5	0,0	2,3	1,8	4	3	3	4	4	10	4	4
29	2	3	1	3	4,8	2,3	5,0	6,5	0,3	6,0	0,3	6,9	7,0	6	3	2	7	7	11	2	7
30	2	3	2	3	5,0	2,0	5,0	7,1	5,0	5,9	0,0	7,0	3,5	13	3	8	12	15	16	9	10
31	2	5	2	2	2,5	1,0	5,0	4,3	0,3	4,8	1,3	3,1	3,0	11	3	6	11	9	14	7	9
32	3	1	1	2	2,5	1,5	5,0	1,3	0,0	7,0	2,8	3,2	5,8	2	3	1	3	2	8	3	2
33	3	1	1	3	4,0	2,3	5,0	11,0	0,0	7,6	0,0	7,1	5,0	18	3	3	13	18	24	10	14
34	1	3	1	2	1,5	0,8	5,0	3,5	5,0	5,5	3,5	3,5	6,0	14	1	6	3	14	17	7	6

ID: Menu sheet identification; **Centre:** Type of University centre (1: University residence; 2: College; 3: University cafeteria); **Region:** Spanish region of the menu sheet (1: Andalucía; 2: Castilla la Mancha; 3: Castilla y León; 4: Galicia; 5: Madrid; 6: País Vasco: 7: Valencia); **Management:** food service management (1: Direct management; 2: Catering management); **Menu options:** options for first and second course (1: unique menu; 2: two options; 3: three or more options).

Table S2. COMUNI questionnaire results of the studied menu sheets.

ID	COMUNI	Item 1	Item 2	Item 3	Item 4	Item 5	Item 6	Item 7	Item 8	Item 9	Item 10	Item 11	Item 12	Item 13	Final score
1	2	1	0	1	0	0	0	0	0	0	1	0	1	0	4
2	2	1	0	1	1	0	0	1	0	0	1	0	1	1	6
3	1	0	0	1	0	0	0	1	0	0	1	0	0	1	3
4	1	0	0	1	0	0	0	1	0	0	1	0	0	0	3
5	2	1	0	1	0	0	0	0	0	0	1	0	0	0	3
6	2	1	0	1	1	1	0	1	0	0	1	0	1	1	7
7	2	0	0	1	1	0	0	0	0	0	1	1	1	1	5
8	1	0	1	1	0	0	1	1	0	0	1	0	1	1	6
9	2	0	0	1	0	1	0	1	0	0	1	0	0	1	4
10	1	0	0	1	0	1	0	1	0	0	1	0	0	0	4
11	1	0	0	0	0	0	0	1	0	0	1	0	0	0	2
12	1	0	1	1	0	0	0	0	0	0	1	0	0	0	3
13	2	0	1	1	0	0	0	1	0	0	1	1	0	1	5
14	1	0	1	1	0	1	0	1	0	0	1	0	0	0	5
15	1	0	0	1	0	0	0	1	0	0	1	0	0	0	3
16	2	1	0	1	1	1	0	1	0	1	1	0	1	1	8
17	2	1	0	1	0	0	0	1	0	0	1	0	1	1	5
18	2	0	0	1	1	0	0	0	0	0	1	0	1	1	4
19	2	0	0	1	0	1	0	0	0	0	1	0	1	1	4
20	2	0	0	1	0	1	0	0	0	0	1	0	0	1	3
21	2	0	0	1	0	1	0	0	0	0	1	0	1	1	4
22	2	0	0	1	0	1	0	0	0	0	1	0	0	1	3
23	2	1	0	1	1	0	0	0	0	0	1	0	0	1	4
24	2	0	0	0	1	0	0	1	0	0	1	0	1	1	4
25	2	1	0	1	1	0	0	0	0	0	1	0	0	1	4
26	1	0	0	1	0	1	0	1	0	0	1	0	0	1	4
27	1	0	0	1	0	1	0	1	0	0	1	0	1	1	5
28	1	0	0	1	0	1	0	1	0	0	1	0	1	1	5
29	2	1	0	1	1	0	0	1	0	0	1	0	0	0	5
30	2	1	0	1	1	1	0	1	0	0	1	0	1	1	7
31	2	0	0	1	0	0	0	0	0	1	1	0	1	1	4
32	2	0	0	1	0	0	0	0	0	0	1	0	0	1	2
33	2	0	0	1	1	0	0	1	0	0	1	0	1	1	5
34	2	0	0	1	0	1	0	0	0	0	1	0	0	0	3

ID: Menu sheet identification; **COMUNI:** Type of COMMUNI questionnaire applied (1: COMUNI-1; 2: COMUNI-2). Each item is scored 1 point if it meets the criterion, or 0 points if it does not.
